# In Vitro Activity of Ceftaroline and Comparators against Bacterial Isolates Collected Globally from Patients with Skin and Soft Tissue Infections: ATLAS Program 2019–2020

**DOI:** 10.3390/antibiotics12081237

**Published:** 2023-07-26

**Authors:** Alona Kuraieva, Guillermo Cabezas-Camarero, Pattarachai Kiratisin, Eric Utt

**Affiliations:** 1 Pfizer Inc., 66 Hudson Blvd. E., New York, NY 10001, USA; 2 Pfizer SLU, 28103 Alcobendas, Madrid, Spain; 3 Department of Microbiology, Faculty of Medicine Siriraj Hospital, Mahidol University, Bangkok 10700, Thailand; 4 Pfizer Inc., Groton, CT 06340, USA

**Keywords:** ceftaroline, antimicrobial activity, antimicrobial resistance, skin and soft tissue infections, methicillin-resistant *Staphylococcus aureus*, surveillance

## Abstract

The objective of this study was to assess the in vitro activity of ceftaroline and a panel of comparator agents against isolates causing skin and soft tissue infections (SSTIs) collected in Africa/Middle East, Asia–Pacific, Europe, and Latin America from 2019–2020. Minimum inhibitory concentrations (MIC) were determined using European Committee on Antimicrobial Susceptibility Testing criteria. All the methicillin-susceptible *Staphylococcus aureus* (MSSA) isolates were susceptible to ceftaroline. Across all regions, ceftaroline demonstrated potent activity against methicillin-resistant *S. aureus* (MRSA, susceptibility 89.5–93.7%) isolates. Susceptibility to vancomycin, daptomycin, linezolid, teicoplanin, trimethoprim sulfamethoxazole, and tigecycline was ≥94.1% in MSSA and MRSA isolates. Against β-hemolytic streptococci isolates, ceftaroline demonstrated very potent activity (MIC_90_ 0.008–0.03 mg/L) across all regions. All β-hemolytic streptococci isolates were susceptible to linezolid, penicillin, and vancomycin (MIC_90_ 0.06–2 mg/L). Among the extended-spectrum β-lactamases (ESBL)-negative Enterobacterales tested (*E. coli*, *K. pneumoniae*, and *K. oxytoca*), susceptibility to ceftaroline was high (88.2–98.6%) in all regions. All ESBL-negative Enterobacterales were susceptible to aztreonam. Potent activity was observed for amikacin, cefepime, and meropenem (94.1–100%) against these isolates. Overall, ceftaroline showed potent in vitro activity against isolates of pathogens causing SSTIs. Continuous surveillance of global and regional susceptibility patterns is needed to guide appropriate treatment options against these pathogens.

## 1. Introduction

Skin and soft tissue infections (SSTIs) are among the major causes of hospitalizations and emergency department visits and are associated with considerable morbidity [1,2]. SSTIs include a range of infections, and the clinical presentation ranges from mild infections such as cellulitis to life-threatening, necrotizing infections of soft tissue [3]. Complicated SSTIs (cSSTIs) require hospitalization and include infections of deeper soft tissues such as necrotizing infections, major abscesses, ulcers, and burns [3,4]. According to the US Food and Drugs Administration (FDA), acute bacterial skin and skin structure infection (ABSSI) includes cellulitis, erysipelas, wound infections, and major cutaneous abscesses with a lesion surface area ≥75 cm^2^ [3]. According to a Global Burden of Disease (GBD) study (1990–2017), there was a 46.8% increase in incidence and a 40.2% increase in years lived with disability for skin and subcutaneous diseases between 1990 and 2017 [5]. A systematic analysis in the GBD study in 2017 estimated a total of 76,000 deaths (48,700–95,600) attributed to bacterial skin diseases with an increase of 45.5% (36.8–54.9%) from 2007–2017 [6]. Another systematic analysis based on data from the GBD database conducted in 2019 revealed an annual increase of 7.38% (7.06–7.67) for age-standardized incidence of bacterial skin disease from 1990–2019 [7].

The major causative organisms of SSTIs are *Staphylococcus aureus* and β-hemolytic streptococci. However, Gram-negative bacilli including *Escherichia coli* and *Klebsiella pneumoniae* are also increasingly involved [8,9,10]. Polymicrobial SSTIs require broad-spectrum antimicrobial treatment and the rise in antimicrobial resistance among the causative organisms is complicating the treatment of SSTIs [8,10,11,12].

Ceftaroline, the active metabolite of the pro-drug ceftaroline fosamil, is a broad-spectrum, parenteral, fifth-generation cephalosporin [13], currently approved by the US FDA for the treatment of adult and pediatric patients with ABSSIs caused by *S. aureus* (including methicillin susceptible [MSSA] and methicillin-resistant [MRSA] isolates), *Streptococcus pyogenes*, *Streptococcus agalactiae*, *E. coli*, *K. pneumoniae*, and *Klebsiella oxytoca* [14]. The European Medicines Agency (EMA) has approved ceftaroline fosamil for the treatment of neonates, infants, children, adolescents, and adults with cSSTIs [15].

The treatment of SSTIs has been complicated by increasing incidence of MRSA which has led to antibiotic misuse [12,16,17,18,19,20,21,22]. Furthermore, in SSTIs caused by drug-resistant Gram-negative bacilli, treatment options are limited [10]. As the incidence of skin infections and antimicrobial resistance is on the rise [5,12,23], it is essential to monitor the global longitudinal trends of antimicrobial susceptibility for ceftaroline and other agents among SSTI isolates for understanding emerging resistance mechanisms to guide appropriate antimicrobial therapy. Previously, a study by Piérard et al. assessed the antimicrobial activity of ceftaroline and comparators in SSTI isolates collected across regions including Africa/Middle East, Asia–Pacific, Europe, and Latin America as part of the Antimicrobial Testing Leadership and Surveillance (ATLAS) surveillance program, from 2015–2018 [24]. Considering that antimicrobial resistance is annually monitored to evaluate the changes in susceptibility patterns of antimicrobials and complications of treatment of SSTIs, we conducted this study with the aim of examining the antimicrobial activity of ceftaroline and a panel of comparator agents against SSTI isolates collected in Africa/Middle East (AfME), Asia–Pacific (APAC), Europe, and Latin America (LATAM) from 2019–2020 as part of the ATLAS program.

## 2. Results

### 2.1. Distribution of Isolates Causing SSTIs

A total of 11,761 isolates of MSSA (n = 5114; 215 sites), MRSA (n = 1824; 194 sites), *S. pyogenes* (n = 723; 157 sites), *Streptococcus agalactiae* (n = 535; 150 sites), *S. dysgalactiae* (n = 202; 117 sites), *E. coli* (n = 1587; 184 sites), *K. pneumoniae* (n = 1505; 184 sites), and *K. oxytoca* (n = 271; 102 sites) were collected from a total of 1085 unique sites in 56 countries located in AfME, APAC, Europe, and LATAM from patients with SSTIs. Approximately half of all isolates were collected in Europe (50.6%, 5962/11,782), followed by APAC (21.9%, 2588/11,782), LATAM (14.6%, 1725/11,782), and AfME (12.8%, 1507/11,782). The numbers of isolates collected in each country and year are presented in Table S1.

### 2.2. Antimicrobial Activity of Ceftaroline and Comparators against Isolates Causing SSTIs

In this study, susceptibility was categorized as high (≥85.0%), moderate (<85.0%), or low (≤60.0%), and antimicrobial activity was categorized, based on susceptibility, as low (≤60.0%), moderate (<85.0%), good (85.0–89.0%), potent (≥89.5%), or very/highly potent (≥95.0%).

#### 2.2.1. MSSA and MRSA

All isolates of MSSA were susceptible to ceftaroline, daptomycin, teicoplanin, and vancomycin in all regions (100%). Across the regions, susceptibility rates were also high for linezolid (99.8–100%), tigecycline (99.7–100%), trimethoprim sulfamethoxazole (97.5–99.8%), clindamycin (93.6–99.1%), and gentamicin (88.3–95.9%). Susceptibility to erythromycin (71.1–81.0%) was moderate in all regions. The MSSA isolates showed high susceptibility to levofloxacin (87.6–95.2%) except in APAC where moderate susceptibility was observed (78.1%) (Table 1).

Among MRSA isolates, susceptibility to ceftaroline was high (89.5–93.7%) in all regions. Additionally, susceptibility to ceftaroline increased with exposure against the MRSA isolates (95–99.6%). Among comparator agents, susceptibility rates were high (≥94.1%) for daptomycin, linezolid, teicoplanin, tigecycline, trimethoprim sulfamethoxazole, and vancomycin across all regions. Regional differences were observed in susceptibility to clindamycin (highest in AfME: 84%, lowest in LATAM: 72.5%), gentamicin (highest in LATAM: 84.7%, lowest in APAC: 55.3%), and erythromycin (highest in AfME: 61.2%, lowest in Europe: 37%). Susceptibility to levofloxacin was highest in LATAM (68.3%) and lowest in Europe (39.1%) (Table 1).

#### 2.2.2. β-Hemolytic Streptococci

Susceptibility data in *S. pyogenes* was limited to small numbers of isolates across AfME and LATAM (n ≤ 97). EUCAST does not publish breakpoints for ceftaroline tested against β-hemolytic streptococci. Hence, only the MIC data are presented. Ceftaroline demonstrated very potent activity (MIC_90_ 0.008–0.015 mg/L) against *S. pyogenes* isolates across all regions. All *S. pyogenes* isolates were susceptible to linezolid, penicillin, tigecycline, and vancomycin. Clindamycin showed highly potent activity (MIC_90_ 0.12 mg/L; susceptibility ≥ 95.6%) across regions, except in APAC (MIC_90_ 2 mg/L; susceptibility 89.7%). Erythromycin showed a similar trend, with potent activity (MIC_90_ 0.06 mg/L; susceptibility ≥ 90.2%) across most regions, except in APAC (MIC_90_ 2 mg/L; susceptibility 76.5%). Levofloxacin demonstrated potent activity (MIC_90_ 1–2 mg/L; susceptibility ≥ 90.7%) in all the regions, with all isolates of *S. pyogenes* collected in AfME being susceptible (Table 2).

Susceptibility data for *S. agalactiae* were limited to small numbers of isolates across AfME and LATAM (n ≤ 74). Ceftaroline demonstrated very potent activity (MIC_90_ 0.03–0.015 mg/L) across all regions. All *S. agalactiae* isolates were susceptible to linezolid, penicillin, tigecycline, and vancomycin in all regions. Antimicrobial activity of clindamycin (MIC_90_ 2 mg/L; susceptibility 77.1–85.1%) and erythromycin (MIC_90_ 2 mg/L; susceptibility 64.6–81.1%) was overall moderate across all regions. Levofloxacin demonstrated potent activity (MIC_90_ 1–2 mg/L; susceptibility ≥ 90.7%) in all the regions (Table 2).

Susceptibility data for *S. dysgalactiae* were limited to small numbers of isolates across most regions (n ≤ 42), except Europe (n = 127). Ceftaroline demonstrated very potent activity (MIC_90_ 0.008–0.015 mg/L) across all regions. All isolates were susceptible to linezolid, penicillin, and vancomycin. The antimicrobial activity of tigecycline was highest in AfME (MIC_90_ 0.12 mg/L; susceptibility 100%) and lowest in APAC (MIC_90_ 0.25 mg/L; susceptibility 85.7%). Clindamycin demonstrated potent activity in AfME (MIC_90_ 0.12 mg/L; susceptibility 92.3%) and LATAM (MIC_90_ 0.5 mg/L; susceptibility 95%), and good activity in Europe (MIC_90_ 2 mg/L; susceptibility 88.2%) and APAC (MIC_90_ 2 mg/L; susceptibility 88.1%). Antimicrobial activity of erythromycin was highest in LATAM (MIC_90_ 2 mg/L; susceptibility 75%) and lowest in AfME (MIC_90_ 2 mg/L; susceptibility 53.9%). Levofloxacin demonstrated very potent activity (MIC_90_ 1 mg/L; susceptibility ≥ 95.0%) in all the regions with all isolates of *S. dysgalactiae* collected in AfME being susceptible (Table 2).

#### 2.2.3. Gram-Negative Enterobacterales

Among 1587 *E. coli* isolates, a total of 909 (57.3%) extended-spectrum β-lactamases (ESBL)-negative *E. coli* isolates were collected. Susceptibility to ceftaroline was high (89.7–93.2%) across all regions. All isolates were susceptible to aztreonam and meropenem. Susceptibility to amikacin, amoxicillin-clavulanate, cefepime, piperacillin–tazobactam, and tigecycline was also high (≥88.1%) across all regions. Susceptibility to levofloxacin and trimethoprim sulfamethoxazole was highest in Europe (80.9% and 73%, respectively) and lowest in LATAM (66% and 55.3%, respectively) (Table 3).

Among 1505 *K. pneumoniae* isolates, there were 613 (40.7%) ESBL-negative *K. pneumoniae* isolates collected during the study, all of which were susceptible to aztreonam. Susceptibility to ceftaroline and piperacillin–tazobactam was lower in Europe (89.60% and 86.40%, respectively) compared with the other three regions (95.20–98.60% and 95.90–96.80%, respectively). Susceptibility to amikacin, amoxicillin–clavulanate, cefepime, and meropenem was high (≥92.40%) across all regions. Susceptibility to levofloxacin and trimethoprim sulfamethoxazole was higher in AfME and APAC (≥90.50% for levofloxacin and ≥87.30% for trimethoprim sulfamethoxazole) compared with Europe and LATAM (≥82.20% for levofloxacin and trimethoprim sulfamethoxazole) (Table 3). 

Among 271 *K. oxytoca* isolates, a total of 208 (76.8%) ESBL-negative *K. oxytoca* isolates were collected, all of which were susceptible to aztreonam and cefepime. Susceptibility to ceftaroline was high across all regions, with lower susceptibility in LATAM (88.2%) compared to other three regions (95–95.5%). Susceptibility to amikacin, amoxicillin–clavulanate, levofloxacin, meropenem, piperacillin–tazobactam, and trimethoprim sulfamethoxazole was high (≥90%) across all regions (Table 3).

## 3. Discussion

This study evaluated the in vitro antimicrobial susceptibilities of ceftaroline and a panel of comparator agents against SSTI isolates collected in AfME, APAC, Europe, and LATAM from 2019–2020. 

In the current study, all MSSA isolates were susceptible to ceftaroline (MIC_90_ 0.25–0.5 mg/L) across all regions. These data are in line with results from two previous global ATLAS studies, one that assessed activity of ceftaroline against SSTI isolates collected in AfME, APAC, Europe, and LATAM from 2015–2018 in which all MSSA isolates were susceptible to ceftaroline (MIC_90_ 0.25–0.5 mg/L) [24] and another study that assessed isolates collected from various infection sources, including SSTIs, from 2012–2017, which reported high susceptibility to ceftaroline (99.9–100%) in MSSA isolates across all regions [25]. These data suggest that ceftaroline is effective against MSSA isolates and its activity has been maintained over the years. Among the comparator agents, vancomycin, daptomycin, teicoplanin, tigecycline, trimethoprim sulfamethoxazole, linezolid, and clindamycin (except for clindamycin in APAC, 93.6%) demonstrated highly potent activity (susceptibility 96.4–100%). These data are consistent with the results from previous global ATLAS studies (2012–2017/2015–2018, susceptibility 95.7–100%/95.9–100%, except clindamycin in APAC, 89.8%/89.2%) [24,25].

Although MRSA isolates in the current study showed high susceptibility (89.5–93.7%) to ceftaroline across all regions, it was slightly lower than that observed in the previous global ATLAS study (2015–2018) [24]. Interestingly, in this study, susceptibility in APAC was slightly lower compared with other regions (APAC vs. other regions, 89.5% vs. 91.6–93.7%). These findings are in line with results from the previous global ATLAS study (2015–2018: 90.8% vs. 93.2–96.5%) [24]. Furthermore, a previous global AWARE surveillance study that included MRSA isolates from cSSTIs collected in 2015–2016 also demonstrated slightly lower susceptibility in Asia (92.2%) compared with Europe and Africa (≥96.2%) [26]. Overall, these findings suggest potent activity for ceftaroline against MRSA isolates, with slightly lower activity in APAC compared with other regions. Importantly, in 2017, the EMA approved a higher dose of ceftaroline (600 mg every 8 h over 120 min) for cSSTI caused by *S. aureus* with an MIC of 2 or 4 mg/L [15]. In line with this, in the current study, ceftaroline demonstrated very potent activity against all MRSA isolates with increased exposure (95–99.6%). The therapeutic options for SSTIs caused by *S. aureus* including MRSA, as recommended by guidelines for SSTIs, include vancomycin, daptomycin, linezolid, teicoplanin, trimethoprim sulfamethoxazole, tigecycline, and clindamycin [4,27,28,29]. Among these, all agents except clindamycin (susceptibility 72.5–84%) demonstrated high susceptibility (94.1–100%) in this study against MRSA isolates. These findings are consistent with the previous global ATLAS studies (2012–2017/2015–2018: ≥89.5%/≥90.9%) [24,25]. Notably, in this study, susceptibility to clindamycin against MRSA isolates was higher across all regions (72.5–84.0%) compared with the previous study (2015–2018: 62.8–72.8%) [24].

In the current study, ceftaroline demonstrated very potent activity against all β-hemolytic streptococci isolates (MIC_90_: *S. pyogenes*, 0.008–0.015 mg/L; *S. agalactiae*, 0.015–0.03 mg/L; *S. dysgalactiae*, 0.008–0.015 mg/L). The activity for ceftaroline against *S. pyogenes* in APAC was lower than other regions (MIC_90_ 0.015 mg/L vs. 0.008 mg/L; n ≤ 97 in AfME and LATAM). Notably, the previous global ATLAS study reported consistent activity for ceftaroline against *S. pyogenes* across all regions (2015–2018: MIC_90_ 0.008 mg/L) [24]. Interestingly, another study including isolates from various infection sources (including SSTIs) from APAC and South Africa during 2011 also reported MIC_90_ ≤0.015 mg/L for *S. pyogenes* [30]. In the current study, the MIC_90_ for ceftaroline against *S. agalactiae* isolates in AfME (n = 48) was 0.03 mg/L, which was higher than the 0.015 mg/L reported previously [24,31,32]. In the current study, the MIC_90_ for ceftaroline against *S. agalactiae* isolates in LATAM (n = 74) was 0.015 mg/L, which was lower than the 0.03 mg/L reported previously by the global ATLAS study (2015–2018) [24]. However, similar to our study, an AWARE study of SSTI isolates collected from LATAM in 2012 reported an MIC_90_ of 0.015 mg/L (CLSI) for ceftaroline in *S. agalactiae* [33]. Among *S. dysgalactiae* isolates collected in Europe, ceftaroline had an MIC_90_ of 0.008 mg/L in the current study. Interestingly, the previous global ATLAS study in 2015–2018 reported a higher MIC_90_ of 0.015 mg/L for ceftaroline against *S. dysgalactiae* isolates in Europe [24]. In the current study, all β-hemolytic streptococci isolates were susceptible to linezolid, penicillin, and vancomycin (MIC_90_ 0.06–2 mg/L), which is supported by previous studies (MIC_90_ ≤ 0.015–2 mg/L) [24,30,31,33,34,35].

Among the ESBL-negative Enterobacterales isolates, ceftaroline demonstrated potent activity against *E. coli* across all regions (MIC_90_ 0.5–1 mg/L). In contrast, the previous global ATLAS study (2015–2018) demonstrated moderate ceftaroline activity in APAC (MIC_90_ 128 mg/L) compared with other regions (MIC_90_ 0.5–4 mg/L) [24]. These data suggest an increase in activity for ceftaroline in APAC compared with previous years. In the current study, ceftaroline demonstrated potent activity against *K. pneumoniae* in Europe (MIC_90_ 1 mg/L) and very potent activity against those collected in APAC (MIC_90_ 0.25 mg/L). Notably, the previous global ATLAS study from 2015–2018 reported moderate activity (MIC_90_ 16 mg/L) for ceftaroline in Europe and APAC [24]. In the current study, ceftaroline demonstrated very potent activity against *K. oxytoca* isolates in Europe (MIC_90_ 0.5 mg/L). In contrast, the global ATLAS study from 2015–2018 reported good activity for ceftaroline against *K. oxytoca* in Europe (MIC_90_ 2 mg/L) [24]. Overall, the results in this study suggest that ceftaroline is potent against ESBL-negative Enterobacterales and could be a good treatment option against these isolates. In the current study, all ESBL-negative Enterobacterales isolates were susceptible to aztreonam (MIC_90_ 0.12–0.5 mg/L). However, the previous global ATLAS study reported variable activity for aztreonam across regions and organisms (MIC_90_ 0.25–32 mg/L) [24]. The current study also reported potent activity for amikacin, cefepime, and meropenem across all regions (MIC_90_ ≤ 0.06–8 mg/L). Interestingly, the previous global ATLAS study noted variation in activity for these agents across regions and organisms (MIC_90_ 0.06–8 mg/L) [24]. 

This study has a few limitations. Approximately half of the isolates collected were from Europe, which could skew the overall data patterns towards those seen in Europe. However, for MRSA, the isolate distribution was well balanced across most regions except AfME. The distribution of participating centers varied across countries and years of the study period, as a result of which all countries and regions are not equally represented within the dataset. A pre-defined number of isolates were collected from each site, so the results of this study cannot be interpreted as prevalence or used for epidemiological data. The low number of samples for some species in this study should be taken into consideration while interpreting the findings. ATLAS is a global surveillance platform focusing on the susceptibility rates of a panel of antimicrobials against clinical isolates from hospitalized patients with various infections including SSTIs, and does not capture biochemical analyses, serology, morbidity and mortality rates, nor clinical outcomes associated with these isolates. Hence, such analyses were not included in this surveillance study which focuses only on the susceptibility rates against pathogens associated with SSTIs. As the current study is an extension of the previous study by Pierard et al. (2015–2018) [24], the number of isolates collected in this study could be lower due to a shorter recruitment period (2019–2020) and the COVID-19 outbreak during 2020. Data for ceftriaxone were unavailable among ESBL-negative Enterobacterales isolates as ceftriaxone was not tested post 2017 in these isolates as part of the ATLAS program. Lastly, tigecycline susceptibility data were unavailable for *K. pneumoniae* and *K. oxytoca* isolates as EUCAST breakpoints for tigecycline are not available for *Klebsiella* species.

In conclusion, this study demonstrates that ceftaroline has potent in vitro activity against Gram-positive and Gram-negative isolates associated with cSSTI collected from AfME, APAC, Europe, and LATAM. Among MRSA isolates, the activity of ceftaroline was slightly lower in APAC compared with other regions, and this trend has been maintained over the years. Among ESBL-negative *E. coli* isolates, an increase in susceptibility to ceftaroline was observed in APAC compared with previous years. Among the comparator agents, our study also identified vancomycin, daptomycin, linezolid, teicoplanin, and tigecycline to be highly active against MSSA and MRSA isolates, whereas linezolid, penicillin, and vancomycin demonstrated very potent activity among β-hemolytic streptococci isolates. Against the Gram-negative isolates, amikacin, aztreonam, cefepime, and meropenem exhibited potent activity. With polymicrobial SSTIs often requiring empirical and broad-spectrum treatment, surveillance of global and regional susceptibility patterns and resistance mechanisms is warranted for ensuring the use of appropriate treatment options and limiting antimicrobial resistance.

## 4. Materials and Methods

### 4.1. Bacterial Isolates

In this study, non-duplicate clinical isolates (single isolate per patient) of *S. aureus*, *S. agalactiae*, *S. dysgalactiae*, *S. pyogenes*, *E. coli*, *K. pneumoniae*, and *K. oxytoca* were collected between 2019–2020 from hospitalized patients with skin and soft tissue infections from AfME, APAC, Europe, and LATAM, as part of ATLAS [36], a global surveillance program implemented in 2004 that provides antimicrobial activity data for different classes of antimicrobials against isolates collected worldwide. North America was not included in this study as this region was not part of the global ATLAS program sponsored by Pfizer. This program collected a predefined set of isolates from each participating center annually, across selected bacterial species and infection types. Isolates were limited to one patient every year and accepted independently of the patient’s hospital location. Bacterial isolates were collected from abscess, bone, burn, carbuncle, cellulitis, decubitus, exudate, furuncle, hair, impetiginous lesions, integumentary (skin, nail, hair), muscle, nails, skin, synovial fluid, tissue fluid, ulcer, wound, other skin, and other skeletal specimen sources. Isolates were shipped to the central laboratory, International Health Management Associates, Inc. (IHMA, Schaumburg, IL, USA) and confirmed using matrix-assisted laser desorption ionization–time-of-flight mass spectrometry (MALDI-TOF MS, Bruker Daltonics, Billerica, MA, USA).

### 4.2. Antimicrobial Susceptibility Testing

Antimicrobial susceptibility testing was performed using broth microdilution methodology for ceftaroline and a panel of comparator antimicrobial agents—amikacin, amoxicillin–clavulanate, aztreonam, cefepime, clindamycin, daptomycin, erythromycin, levofloxacin, linezolid, meropenem, piperacillin–tazobactam, penicillin, tigecycline, trimethoprim sulfamethoxazole, and vancomycin, according to Clinical and Laboratory Standards Institute (CLSI) guidelines [37]. Minimum inhibitory concentrations (MICs) were interpreted using European Committee on Antimicrobial Susceptibility Testing (EUCAST) breakpoints (version 12.0) [38]. All tests were conducted using the appropriate quality control strains from the American Type Culture Collection (ATCC) following CLSI guidelines. The results were included in the analysis only when corresponding quality control isolates were within the acceptable ranges according to CLSI guidelines. An extensive array of electronic expert analysis algorithms was utilized by IHMA to analyze all tested results for quality assurance (QA). Those results that did not pass the QA were further reviewed by microbiologists (PhD) for evaluability. The QC strains ensured the accuracy of the testing to identify susceptible and resistant isolates in the set of isolates tested. The isolates from China were identified and tested by a central lab in China. Methicillin resistance for each *S. aureus* isolate was determined using the oxacillin MIC method (MIC ≥ 4 mg/L, confirmed methicillin resistance). MIC breakpoints for ceftaroline in β-hemolytic streptococci have not been defined by EUCAST, specifying that the susceptibility to ceftaroline can be inferred from testing benzyl penicillin [38]. Resistance phenotypes of β-hemolytic streptococci are not available on the ATLAS database; hence they were not included in the study. Isolates of *E. coli*, *K. pneumoniae*, and *K. oxytoca* with a ceftazidime or aztreonam MIC ≥ 2 mg/L were screened for the presence of extended-spectrum β-lactamases (ESBL) genes—*bla*_SHV_, *bla*_TEM_, *bla*_CTX-M_, *bla*_VEB_, *bla*_PER_, and *bla*_GES_, using multiplex PCR assays followed by full-gene DNA sequencing as previously described [39]. ESBL-negative isolates were defined as those for which a gene encoding an ESBL was not detected and those that did not meet the criteria for molecular screening (MIC < 2 to both ceftazidime and aztreonam). Ceftaroline is known to be inactive against most isolates of Enterobacterales carrying ESBLs, so the current study focused on non-ESBL-producing isolates of Enterobacterales. Data for ceftriaxone were not tested post 2017 among non-ESBL-producing Enterobacterales, so ceftriaxone activity was not evaluated in these isolates.

All the data were collected and presented as a percentage of susceptible (%S) isolates, MIC_90_, and an MIC range based on EUCAST guidelines for all identified organisms. In this study, susceptibility was categorized as high (≥85.0%), moderate (<85.0%), or low (≤60.0%), and antimicrobial activity was categorized, based on susceptibility, as low (≤60.0%), moderate (<85.0%), good (85.0–89.0%), potent (≥89.5%), or very/highly potent (≥95.0%). No statistical analysis was performed as part of this study.

## Figures and Tables

**Table 1:** In vitro antimicrobial activity of ceftaroline and comparators against methicillin-susceptible *Staphylococcus aureus* (MSSA) and methicillin-resistant *S. aureus* (MRSA) isolates collected from skin and soft tissue infections (SSTIs) (2019–2020).

Organism/ Antimicrobial	Africa/Middle East			Asia-Pacific			Europe			Latin America			
	MIC_90_ (mg/L)	MIC Range (mg/L)	%S	MIC_90_ (mg/L)	MIC Range (mg/L)	%S	MIC_90_ (mg/L)	MIC Range (mg/L)	%S	MIC_90_ (mg/L)	MIC Range (mg/L)	%S	Susceptibility (%S)/Activity Attributes
MSSA	n = 629			n = 1122			n = 2775			n = 588			
Ceftaroline	0.25	0.06–0.5	100	0.5	0.06–1	100	0.25	0.06–1	100	0.5	0.06–0.5	100	High %S/ very/highly potent
Clindamycin	0.12	0.03–4	99.1	0.25	0.03–4	93.6	0.12	0.03–4	97.0	0.12	0.03–4	96.4	High %S/ potent to very/highly potent
Daptomycin	1	0.06–1	100	1	0.06–4	99.7	1	0.06–4	99.3	1	0.12–2	99.8	High %S/ very/highly potent
Erythromycin	8	0.12–8	78.2	8	0.12–8	71.1	8	0.12–8	81.0	8	0.12–8	71.1	Moderate
Gentamicin	1	1–32	94.4	4	1–32	88.8	1	1–32	95.9	4	1–32	88.3	High %S/ good to very/highly potent
Levofloxacin ^a^	4	0.03–8	87.6	8	0.06–8	78.1	0.5	0.03–8	94.9	0.5	0.06–8	95.2	Moderate to high %S/ moderate to very/highly potent
Linezolid	2	0.5–16	99.8	2	0.5–4	100	2	0.5–4	100	2	0.5–4	100	High %S/ very/highly potent
Teicoplanin	1	0.12–2	100	1	0.25–2	100	1	0.12–2	100	1	0.25–2	100	High %S/ very/highly potent
Tigecycline	0.12	0.015–1	99.8	0.25	0.015–1	99.7	0.12	0.015–2	99.9	0.12	0.03–0.25	100	High %S/ very/highly potent
Trimethoprim sulfamethoxazole ^b^	0.5	0.03–4	97.5	0.5	0.03–4	98.2	0.12	0.03–4	99.6	0.12	0.03–4	99.8	High %S/ very/highly potent
Vancomycin	1	0.25–2	100	1	0.5–2	100	1	0.25–2	100	1	0.5–2	100	High %S/ very/highly potent
MRSA	n = 237			n = 494			n = 655			n = 438			
Ceftaroline ^c^	1	0.25–4	93.7	2	0.25–8	89.5	1	0.25–32	92.5	1	0.25–4	91.6	High %S/ potent
Clindamycin	4	0.03–4	84.0	4	0.06–4	75.9	4	0.03–4	72.5	4	0.03–4	74.9	Moderate
Daptomycin	1	0.25–1	100	1	0.12–1	100	1	0.12–8	98.0	1	0.25–1	100	High %S/ very/highly potent
Erythromycin	8	0.25–8	61.2	8	0.12–8	37.7	8	0.12–8	37.0	8	0.12–8	42.5	Low
Gentamicin	32	1–32	66.7	32	1–32	55.3	32	1–32	83.1	32	1–32	84.7	Low to moderate
Levofloxacin ^a^	8	0.12–8	54.0	8	0.12–8	39.5	8	0.12–8	39.1	8	0.12–8	68.3	Low to moderate
Linezolid	2	1–4	100	2	1–16	99.8	2	0.5–4	100	2	1–4	100	High %S/ very/highly potent
Teicoplanin	1	0.12–2	100	1	0.25–8	96.2	1	0.12–16	99.1	1	0.25–2	100	High %S/ very/highly potent
Tigecycline	0.12	0.03–0.25	100	0.25	0.03–1	98.6	0.25	0.03–2	99.9	0.25	0.03–1	99.5	High %S/ very/highly potent
Trimethoprim sulfamethoxazole ^b^	2	0.03–4	94.1	0.5	0.03–4	96.4	0.12	0.03–4	98.5	0.12	0.03–4	99.5	High %S/ potent to very/highly potent
Vancomycin	1	0.5–2	100	1	0.5–2	100	2	0.5–8	99.9	2	0.5–2	100	High %S/ very/highly potent

^a^ For levofloxacin, the EUCAST interpretation is susceptibility, increased exposure (SIE). ^b^ Trimethoprim sulfamethoxazole %SIE—MSSA: AfME—2.5%, APAC—1.8%; MRSA: AfME—5.9%, APAC—3.6%, Europe—1.5%. ^c^ Ceftaroline %SIE—MRSA: AfME—5.9%, APAC—5.5%, Europe—7%, LATAM—8%. AfME, Africa/Middle East; APAC, Asia–Pacific; LATAM, Latin America; MSSA, methicillin-susceptible *S. aureus*; n, number of isolates tested; %S, susceptible; SSTI, skin and soft tissue infections.

**Table 2:** In vitro antimicrobial activity of ceftaroline and comparators against β-hemolytic streptococci isolates collected from SSTIs (2019–2020).

Organism/ Antimicrobial	Africa/Middle East			Asia-Pacific			Europe			Latin America			
	MIC_90_ (mg/L)	MIC Range (mg/L)	%S	MIC_90_ (mg/L)	MIC Range (mg/L)	%S	MIC_90_ (mg/L)	MIC Range (mg/L)	%S	MIC_90_ (mg/L)	MIC Range (mg/L)	%S	Susceptibility (%S)/Activity Attributes
*Streptococcus pyogenes*	n = 62			n = 136			n = 428			n = 97			
Ceftaroline ^a^	0.008	0.004–0.015	NA	0.015	0.004–0.03	NA	0.008	0.004–0.12	NA	0.008	0.004–0.03	NA	Very/highly potent
Clindamycin	0.12	0.015–2	98.4	2	0.03–2	89.7	0.12	0.015–2	95.6	0.12	0.015–2	97.9	High %S/ Potent to very/highly potent
Daptomycin ^b^	NA	NA	NA	NA	NA	NA	NA	NA	NA	NA	NA	NA	
Erythromycin ^c^	0.06	0.015–2	93.6	2	0.015–2	76.5	0.25	0.015–2	90.2	0.06	0.015–2	92.8	Moderate to high %S/ Moderate to potent
Levofloxacin ^d^	1	0.25–2	100	1	0.25–8	98.5	1	0.25–8	98.8	2	0.25–8	90.7	High %S/ Potent to very/highly potent
Linezolid	2	0.5–2	100	2	0.5–2	100	2	0.12–2	100	2	0.5–2	100	High %S/ Very/highly potent
Penicillin	0.06	0.06–0.06	100	0.06	0.06–0.12	100	0.06	0.06–0.12	100	0.06	0.06–0.06	100	High %S/ Very/highly potent
Tigecycline	0.06	0.015–0.06	100	0.06	0.008–0.06	100	0.06	0.015–0.06	100	0.06	0.015–0.06	100	High %S/ Very/highly potent
Vancomycin	1	0.25–1	100	0.5	0.12–1	100	0.5	0.03–1	100	1	0.25–1	100	High %S/ Very/highly potent
*Streptococcus agalactiae*	n = 48			n = 105			n = 308			n = 74			
Ceftaroline ^a^	0.03	0.008–0.03	NA	0.03	0.004–0.06	NA	0.015	0.004–0.03	NA	0.015	0.004–0.06	NA	Very/highly potent
Clindamycin	2	0.03–2	81.3	2	0.03–2	77.1	2	0.015–2	78.3	2	0.015–2	85.1	Moderate to high %S/ Moderate to good
Daptomycin ^b^	NA	NA	NA	NA	NA	NA	NA	NA	NA	NA	NA	NA	
Erythromycin	2	0.03–2	64.6	2	0.03–2	69.5	2	0.015–2	69.5	2	0.015–2	81.1	Moderate
Levofloxacin ^d^	1	0.5–8	97.9	2	0.25–8	90.5	2	0.25–8	98.1	2	0.25–8	94.6	High %S/ Potent to very/highly potent
Linezolid	2	0.25–2	100	2	0.5–2	100	2	0.12–2	100	2	0.5–2	100	High %S/ Very/highly potent
Penicillin	0.06	0.06–0.12	100	0.06	0.06–0.12	100	0.06	0.06–0.12	100	0.06	0.06–0.25	100	High %S/ Very/highly potent
Tigecycline	0.06	0.03–0.12	100	0.06	0.015–0.12	100	0.06	0.015–0.06	100	0.06	0.015–0.06	100	High %S/ Very/highly potent
Vancomycin	0.5	0.12–1	100	0.5	0.25–1	100	0.5	0.03–1	100	0.5	0.25–1	100	High %S/ Very/highly potent
*Streptococcus dysgalactiae*	n = 13			n = 42			n = 127			n = 20			
Ceftaroline ^a^	0.015	0.004–0.015	NA	0.015	0.004–0.03	NA	0.008	0.004–0.015	NA	0.015	0.004–0.25	NA	Very/highly potent
Clindamycin	0.12	0.06–2	92.3	2	0.03–2	88.1	2	0.03–2	88.2	0.5	0.06–2	95	High %S/ Good to very/highly potent
Daptomycin ^b^	NA	NA	NA	NA	NA	NA	NA	NA	NA	NA	NA	NA	
Erythromycin ^c^	2	0.03–2	53.9	2	0.015–2	64.3	2	0.03–2	72.4	2	0.03–2	75	Low to moderate
Levofloxacin ^d^	1	0.25–1	100	1	0.25–8	97.6	1	0.25–8	99.2	1	0.25–8	95	High %S/ Very/highly potent
Linezolid	2	1–2	100	2	0.5–2	100	2	0.5–2	100	2	1–2	100	High %S/ Very/highly potent
Penicillin	0.06	0.06–0.06	100	0.06	0.06–0.06	100	0.06	0.06–0.06	100	0.06	0.06–0.06	100	High %S/ Very/highly potent
Tigecycline	0.12	0.03–0.12	100	0.25	0.03–0.25	85.7	0.06	0.015–0.25	98.4	0.25	0.03–0.25	90	High %S/ Good to very/highly potent
Vancomycin	0.5	0.25–0.5	100	0.5	0.25–1	100	0.5	0.25–1	100	0.5	0.25–0.5	100	High %S/ Very/highly potent

^a^ EUCAST does not publish breakpoints for ceftaroline against β-hemolytic streptococci, and states that susceptibility to ceftaroline can be inferred from testing benzyl penicillin. ^b^ Data for daptomycin were not available across all regions. ^c^ Susceptibility with increased exposure (SIE) to erythromycin: *S. pyogenes*: AfME—1.6%; *S. dysgalactiae*: APAC—4.8%. ^d^ For levofloxacin, the EUCAST interpretation is susceptibility, increased exposure (SIE). AfME, Africa/Middle East; APAC, Asia–Pacific; LATAM, Latin America; n, number of isolates tested; NA, not available; %S, susceptible; SSTI, skin and soft tissue infections.

**Table 3:** In vitro antimicrobial activity of ceftaroline and comparators against Gram-negative isolates collected from SSTIs (2019–2020).

Organism/ Antimicrobial	Africa/Middle East			Asia-Pacific			Europe			Latin America			
	MIC_90_ (mg/L)	MIC Range (mg/L)	%S	MIC_90_ (mg/L)	MIC Range (mg/L)	%S	MIC_90_ (mg/L)	MIC Range (mg/L)	%S	MIC_90_ (mg/L)	MIC Range (mg/L)	%S	Susceptibility (%S)/Activity Attributes
*Escherichia coli*, ESBL-negative	n = 109			n = 126			n = 571			n = 103			
Ceftaroline	0.5	≤0.015–≥16	92.7	1	0.03–≥16	89.7	0.5	≤0.015–≥16	93	0.5	≤0.015–≥16	93.2	High %S/ potent
Amikacin	4	1–32	97.2	4	1–16	99.2	4	≤0.25–≥128	98.2	8	1–16	99	High %S/ very/highly potent
Amoxicillin-clavulanate	16	0.25–≥32	89	16	1–≥32	88.1	8	0.25–≥32	93	8	0.5–≥32	93.2	High %S/ good to potent
Aztreonam	0.12	≤0.015–1	100	0.25	0.03–1	100	0.12	≤0.015–1	100	0.12	≤0.015–1	100	High %S/ very/highly potent
Cefepime ^a^	≤0.12	≤0.12–2	99.1	≤0.12	≤0.12–1	100	≤0.12	≤0.12–16	98.8	≤0.12	≤0.12–8	98.1	High %S/ very/highly potent
Levofloxacin ^b^	≥16	≤0.25–≥16	71.6	≥16	≤0.25–≥16	69.8	≥16	≤0.25–≥16	80.9	≥16	≤0.25–≥16	66	Moderate
Meropenem	≤0.06	≤0.06–≤0.06	100	≤0.06	≤0.06–2	100	≤0.06	≤0.06–0.5	100	≤0.06	≤0.06–0.25	100	High %S/ very/highly potent
Piperacillin-tazobactam	4	0.5–≥128	93.6	8	0.5–≥128	91.3	4	≤0.12–≥128	94.7	8	0.25–≥128	94.2	High %S/ potent
Tigecycline	0.25	0.06–2	98.2	0.5	0.06–2	96.8	0.25	≤0.03–2	98.9	0.25	0.06–2	99	High %S/ very/highly potent
Trimethoprim sulfamethoxazole ^c^	≥64	1–≥64	57.8	≥64	1–≥64	62.7	≥64	1–≥64	73	≥64	1–≥64	55.3	Low to moderate
*Klebsiella pneumoniae*, ESBL-negative	n = 63			n = 160			n = 317			n = 73			
Ceftaroline	0.25	0.03–1	95.2	0.25	0.03–≥16	98.1	1	≤0.015–≥16	89.6	0.25	0.03–2	98.6	High %S/ potent to very/highly potent
Amikacin	2	0.5–4	100	2	≤0.25–16	99.4	2	≤0.25–32	98.7	2	0.5–64	98.6	High %S/ very/highly potent
Amoxicillin-clavulanate	4	0.5–8	100	4	0.25–≥32	96.9	8	0.25–≥32	92.4	4	0.5–16	97.3	High %S/ potent to very/highly potent
Aztreonam	0.12	≤0.015–0.25	100	0.12	≤0.015–1	100	0.25	≤0.015–1	100	0.12	≤0.015–0.5	100	High %S/ very/highly potent
Cefepime ^a^	≤0.12	≤0.12–0.25	100	≤0.12	≤0.12–16	99.4	0.25	≤0.12–16	98.1	≤0.12	≤0.12–8	98.6	High %S/ very/highly potent
Levofloxacin ^b^	0.5	≤0.25–≥16	90.5	0.5	≤0.25–≥16	92.5	2	≤0.25–≥16	83.6	1	≤0.25–≥16	82.2	Moderate to high %S/ moderate to potent
Meropenem	≤0.06	≤0.06–≤0.06	100	≤0.06	≤0.06–0.12	100	≤0.06	≤0.06–≥32	99.4	≤0.06	≤0.06–0.5	100	High %S/ very/highly potent
Piperacillin-tazobactam	4	1–≥128	96.8	4	≤0.5–≥128	96.3	16	≤0.5–≥128	86.4	4	≤0.5–32	95.9	High %S/ good to very/highly potent
Tigecycline ^d^	0.5	0.12–2	NA	1	0.06–4	NA	1	0.06–4	NA	1	0.25–4	NA	Very/highly potent
Trimethoprim sulfamethoxazole ^c^	≥64	1–≥64	87.3	4	1–≥64	88.8	≥64	1–≥64	83.9	≥64	1–≥64	82.2	Moderate to high %S/ moderate to good
*Klebsiella oxytoca*, ESBL-negative	n = 20			n = 22			n = 149			n = 17			
Ceftaroline	0.5	0.06–1	95	0.5	≤0.015–1	95.5	0.5	0.03–≥16	95.3	1	0.03–1	88.2	High %S/ good to very/highly potent
Amikacin	2	1–4	100	2	1–2	100	2	≤0.25–8	100	4	1–16	94.1	High %S/ potent to very/highly potent
Amoxicillin-clavulanate	4	1–≥32	90	4	1–≥32	95.5	2	≤0.12–≥32	98	4	1–≥32	94.1	High %S/ potent to very/highly potent
Aztreonam	0.5	≤0.015–0.5	100	0.5	≤0.015–1	100	0.5	≤0.015–1	100	0.25	≤0.015–0.25	100	High %S/ very/highly potent
Cefepime	≤0.12	≤0.12–≤0.12	100	≤0.12	≤0.12–≤0.12	100	≤0.12	≤0.12–0.5	100	≤0.12	≤0.12–1	100	High %S/ very/highly potent
Levofloxacin ^b^	≤0.25	≤0.25–≤0.25	100	≤0.25	≤0.25–≤0.25	100	≤0.25	≤0.25–≥16	98	0.5	≤0.25–0.5	100	High %S/ very/highly potent
Meropenem	≤0.06	≤0.06–≤0.06	100	≤0.06	≤0.06–≤0.06	100	≤0.06	≤0.06–4	99.3	≤0.06	≤0.06–0.12	100	High %S/ very/highly potent
Piperacillin-tazobactam	4	0.5–8	100	4	0.5–4	100	4	0.25–≥128	97.3	4	0.5–4	100	High %S/ very/highly potent
Tigecycline ^d^	0.5	0.12–0.5	NA	0.5	0.06–0.5	NA	0.5	0.06–4	NA	0.5	0.12–1	NA	Very/highly potent
Trimethoprim sulfamethoxazole	1	1–1	100	1	1–≥64	95.5	1	1–≥64	98.7	1	1–≥64	94.1	High %S/ potent to very/highly potent

^a^ Cefepime % susceptible with increased exposure (SIE)—*E. coli*: LATAM—1%; *K. pneumoniae*: Europe—1.6%. ^b^ Levofloxacin %SIE—*E. coli*: APAC—4%, Europe—1.6%, LATAM—3.9%; *K. pneumoniae*: AfME—6.3%, APAC—2.5%, Europe—5.4%, LATAM—9.6%; *K. oxytoca*: Europe—1.3%. ^c^ Trimethoprim sulfamethoxazole %SIE—*E. coli*: LATAM—1%; *K. pneumoniae*: APAC—1.3%, Europe—1.9%. ^d^ EUCAST breakpoints for tigecycline are not available for *K. pneumoniae* and *K. oxytoca*. AfME, Africa/Middle East; APAC, Asia–Pacific; ESBL, extended-spectrum β-lactamases; LATAM, Latin America; n, number of isolates tested; NA, not available; SSTI, skin and soft tissue infections; %S, susceptible; SSTI, skin and soft tissue infections.

## Data Availability

Data are available in a publicly accessible repository that does not issue DOIs. Publicly available datasets were analyzed in this study. These data can be found here: [https://atlas-surveillance.com] (accessed on 2 December 2022).

## Supplementary Materials

The following supporting information can be downloaded at: https://www.mdpi.com/article/10.3390/antibiotics12081237/s1, Table S1: List of countries contributing isolates collected from SSTIs (2019–2020).
