# Supplementary material for: In Vitro Activity of Ceftaroline and Comparators against Bacterial Isolates Collected Globally from Patients with Skin and Soft Tissue Infections: ATLAS Program 2019–2020
Source: Antibiotics (Basel). 2023 Jul 26;12(8):1237. doi: 10.3390/antibiotics12081237 (PMC10451970; doi:10.3390/antibiotics12081237)
Supplement: Supplementary file 1 [file antibiotics-12-01237-s001.zip › antibiotics-2413725-supplementary.pdf]

antibiotics

MDPI

Table S1. List of countries contributing isolates collected from SSTIs (2019–2020).

		Contributing sites and number of isolates by year					
**Region**	**Contributing countries**	**2019**		**2020**		**2019–2020**	
		**Contributing Sites[a]**	**Number of isolates**	**Contributing Sites[a]**	**Number of isolates**	**Contributing Sites[a]**	**Number of isolates**
**Africa/Middle East**		**85**	**691**	**90**	**816**	**110**	**1507**
	Cameroon	0	0	3	10	3	10
	Israel	20	144	17	179	24	323
	Ivory Coast	0	0	3	17	3	17
	Jordan	2	5	1	1	2	6
	Kuwait	10	137	17	161	17	298
	Morocco	16	98	14	102	19	197
	Nigeria	10	93	9	120	10	213
	Qatar	3	16	5	31	5	47
	Saudi Arabia	5	51	4	44	5	95
	South Africa	19	147	17	151	22	294
**Asia-Pacific**		**194**	**1248**	**161**	**1340**	**236**	**2588**
	China	42	253	14	92	50	345
	Hong Kong	5	46	7	70	9	116
	India	38	285	29	338	39	623
	Japan	8	31	12	59	14	90
	Korea, South	7	46	9	78	12	124
	Malaysia	12	54	9	75	12	129
	Philippines	17	102	14	122	19	224
	Taiwan	18	138	14	75	20	213

	Thailand	19	79	17	120	22	199
	Australia	25	179	26	235	28	414
	New Zealand	3	35	10	76	11	111
**Europe**		**487**	**3201**	**441**	**2761**	**598**	**5962**
	Belgium	30	158	32	154	44	312
	Croatia	13	65	15	82	18	147
	Czech Republic	21	156	21	144	24	300
	Denmark	7	40	1	6	7	46
	Finland	6	48	7	73	7	121
	France	36	174	41	171	49	345
	Germany	61	404	51	366	71	770
	Greece	14	106	22	146	24	252
	Hungary	22	132	27	175	27	307
	Ireland	13	101	9	74	14	175
	Italy	37	220	30	208	41	428
	Latvia	4	17	5	16	5	33
	Lithuania	13	44	11	56	14	100
	Netherlands	9	55	9	47	10	102
	Poland	23	119	20	108	24	227
	Portugal	20	125	12	111	20	236
	Romania	11	71	12	100	16	171
	Russia	35	396	1	4	35	396
	Slovenia	0	0	11	40	7	36
	Spain	62	434	56	344	71	784
	Sweden	4	46	4	16	5	57
	Switzerland	8	56	4	32	10	95
	Turkey	14	54	14	62	17	116
	Ukraine	4	43	14	129	14	172

	United Kingdom	20	137	12	97	24	234
**Latin America**		**119**	**810**	**128**	**792**	**156**	**1725**
	Argentina	12	82	15	129	17	222
	Brazil	12	60	25	88	26	174
	Chile	15	72	13	56	16	131
	Colombia	22	129	14	86	26	221
	Costa Rica	5	33	5	25	6	63
	Dominican Republic	4	39	5	43	5	88
	Guatemala	12	76	12	89	14	196
	Mexico	23	179	24	145	28	347
	Panama	5	32	8	54	9	92
	Venezuela	9	108	7	77	9	191

[a] Indicates number of unique sites that collected isolates.
